# KRAS and RAS-MAPK Pathway Deregulation in Mature B Cell Lymphoproliferative Disorders

**DOI:** 10.3390/cancers14030666

**Published:** 2022-01-28

**Authors:** Elena Vendramini, Riccardo Bomben, Federico Pozzo, Tamara Bittolo, Erika Tissino, Valter Gattei, Antonella Zucchetto

**Affiliations:** Clinical and Experimental Onco-Hematology Unit, Centro di Riferimento Oncologico di Aviano (CRO) IRCCS, 33081 Aviano, Italy; rbomben@cro.it (R.B.); federico.pozzo@cro.it (F.P.); tamara.bittolo@cro.it (T.B.); etissino@cro.it (E.T.); vgattei@cro.it (V.G.); zucchetto.soecs@cro.it (A.Z.)

**Keywords:** KRAS, RAS-MAPK pathway, RAS/RAF/MEK/ERK inhibitors, mature B cell lymphoproliferative disorders

## Abstract

**Simple Summary:**

*KRAS* and genes in the RAS-MAPK pathway are among the most frequently deregulated genes in solid tumors and in this context a large amount of KRAS and RAS-MAPK targeting molecules have been developed and tested. The improved understanding of genomic variation in mature B cell neoplasms revealed a considerable portion of patients presenting with KRAS or RAS-MAPK pathway aberrations. These patients could potentially benefit from the use of RAS-RAF-MEK-ERK inhibitors, and in most of the cases, clinical investigation is only in the beginning. Here, we review the incidence of KRAS and RAS-MAPK mutations in mature B cell lymphoproliferative disorders, their association with progression and survival, and current therapeutic strategies targeting RAS-MAPK signaling.

**Abstract:**

*KRAS* mutations account for the most frequent mutations in human cancers, and are generally correlated with disease aggressiveness, poor prognosis, and poor response to therapies. KRAS is required for adult hematopoiesis and plays a key role in B cell development and mature B cell proliferation and survival, proved to be critical for B cell receptor-induced ERK pathway activation. In mature B cell neoplasms, commonly seen in adults, KRAS and RAS-MAPK pathway aberrations occur in a relevant fraction of patients, reaching high recurrence in some specific subtypes like multiple myeloma and hairy cell leukemia. As inhibitors targeting the RAS-MAPK pathway are being developed and improved, it is of outmost importance to precisely identify all subgroups of patients that could potentially benefit from their use. Herein, we review the role of KRAS and RAS-MAPK signaling in malignant hematopoiesis, focusing on mature B cell lymphoproliferative disorders. We discuss KRAS and RAS-MAPK pathway aberrations describing type, incidence, mutual exclusion with other genetic abnormalities, and association with prognosis. We review the current therapeutic strategies applied in mature B cell neoplasms to counteract RAS-MAPK signaling in pre-clinical and clinical studies, including most promising combination therapies. We finally present an overview of genetically engineered mouse models bearing KRAS and RAS-MAPK pathway aberrations in the hematopoietic compartment, which are valuable tools in the understanding of cancer biology and etiology.

## 1. Introduction

The RAS-MAPK pathway is one of the most deregulated and extensively characterized pathways in human cancer, with *KRAS* being the most frequently mutated gene. The RAS-MAPK pathway plays a crucial role in the control of cell proliferation, differentiation and survival, and its aberrant activation in the oncogenic context is frequently associated with invasion, transformation, disease aggressiveness and drug resistance [1].

Inhibitory molecules, targeting single or multiple components of the RAS-MAPK signaling, have been developed and made available for clinical use. Many RAS-MAPK inhibitors are being evaluated in clinical trials with encouraging results, although the extent and duration of efficacy differ depending on tumor type and pattern of genetic aberrations. As strategies to counteract aberrant RAS-MAPK signaling keep improving, such as by the combinatorial use of multiple agents, it is of the utmost importance to precisely identify all subgroups of patients that could potentially benefit from their use.

The first evidence of *RAS* mutations in mature B cell malignancies date back to 1989, when Neri and colleagues identified *KRAS* and *NRAS* mutations in a subgroup of multiple myeloma cases [2]. But it is only with the advent of high-throughput sequencing techniques that the mutational landscape of mature B cell neoplasms has been disclosed, revealing its extreme heterogeneity. Intensive investigation approaches based on whole-genome and whole-exome sequencing (WGS/WES) and the contextual analysis of paired normal samples allowed the identification of a broad spectrum of aberrations. So far, aberrations in genes that are part of the RAS-MAPK signaling have been identified in most of the mature B cell neoplasms, but they present with very different occurrence, and are associated to distinct biological and clinical significance.

In this review we outline the incidence and the main features of KRAS and RAS-MAPK aberrations in the context of mature B cell malignancies. We discuss the main clinical and pre-clinical therapeutic approaches to counteract aberrant RAS-MAPK signaling in the different B cell neoplasms. Finally, we present an overview of conditional mouse models that through expression of RAS-MAPK aberrations in different hematopoietic compartments may help in the understanding of their oncogenic role.

## 2. KRAS and RAS-MAPK Pathway in B Lymphocytes

### 2.1. RAS-MAPK Signaling

RAS proteins are ubiquitously expressed membrane-bound small GTPases that, transducing signals from cell surface receptors to intracellular effector pathways, modulate numerous cellular processes including cell growth, differentiation, and survival. In humans, three *RAS* genes, *KRAS*, *NRAS* and *HRAS,* encode for four RAS proteins, namely KRAS4A, KRAS4B, NRAS and HRAS. These four isoforms are highly homologous and differ mostly in the C-terminal region, relevant for RAS protein maturation, membrane binding and function. RAS proteins act as molecular switches and cycles between an inactive GDP-bound state and an active GTP-bound state. RAS activity is regulated by two groups of proteins, the guanine nucleotide exchange factors (GEFs) and the GTPase activating proteins (GAPs). GEFs, such as son of sevenless (SOS) and Ras guanyl nucleotide releasing proteins (RasGRPs), promote GDP to GTP exchange, leading to GTP-bound RAS active state. GAPs, such as NF1 and RasGAP, stimulate the RAS GTPase activity promoting the switch back to GDP-bound RAS inactive state [1]. 

Among the numerous RAS activated effector pathways, the RAF-MEK-ERK (MAPK, mitogen-activated protein kinase) pathway is the one best characterized and more strongly involved in human cancer. RAF is a family of serine/threonine kinases comprising ARAF, BRAF and CRAF/RAF1. RAS activation promotes cytosolic RAF recruitment to plasma membrane, dimerization and phosphorylation. Activated RAF phosphorylates MEK, which in turn phosphorylates ERK. Activated ERK phosphorylates hundreds of targets in the cytoplasm and nucleus regulating many cellular processes including growth, proliferation and survival. Among RAF proteins, BRAF is the prevalent isoform, being more easily activated by RAS and having higher basal kinase activity. However, increasing attention is arising around RAF1 and its regulatory role on RAF kinases, as observed in BRAF inhibitor-mediated paradoxical ERK activation [3]. 

Another well-known RAS effector is the phosphatidylinositol 3 kinase (PI3K) pathway that is activated through active RAS binding to the p110 catalytic subunit and the enhancing of its catalytic activity [4]. 

### 2.2. Role of the BCR Induced RAS-MAPK Pathway Activation in B Cells

The BCR guides and controls every stages of B cell life. The BCR is a surface membrane molecular complex composed of a pair of identical immunoglobulin heavy (IgH) and light (IgL) chains whose unique variable region determines the specificity for antigen recognition, associated to the heterodimer of signal transduction subunits CD79A (Igα) and CD79B (Igβ) that allow the transduction of BCR signals. 

**Figure 1:**
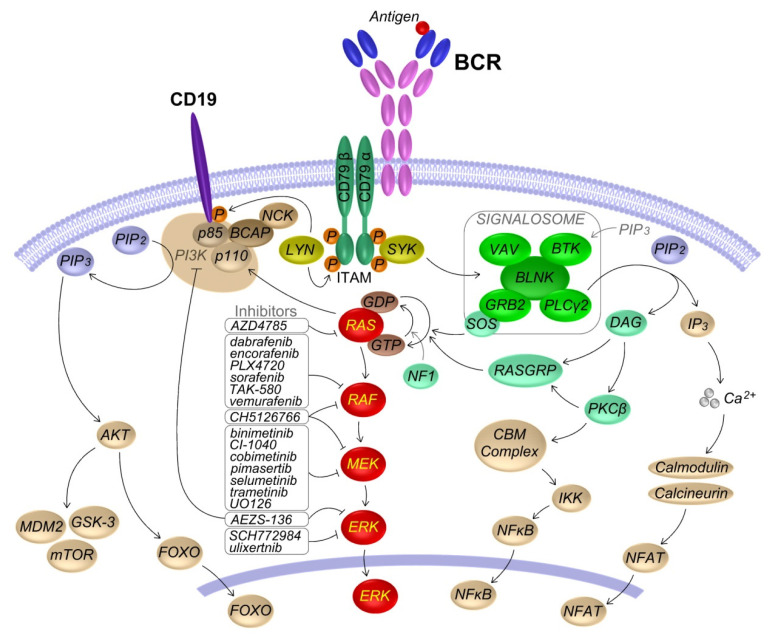
BCR signaling, RAS-MAPK pathway and RAS-MAPK inhibitors. Upon B cell receptor (BCR) antigen binding, LYN (or other SRC kinases) phosphorylates the ITAM domain in the signal transduction subunits CD79A/B. This event promotes the recruitment and activation of spleen tyrosine kinase (SYK), phosphorylation of BLNK and formation of the signalosome. SYK activation of proteins in the signalosome, namely Bruton’s tyrosine kinase (BTK), phospholipase Cγ2 (PLCγ2), VAV and the growth factor receptor bound protein 2 (GRB2), eventually results in the activation of the nuclear factor of activated T cells (NFAT), the nuclear factor κ B (NFκB) and the RAS-MAPK signaling pathways. LYN phosphorylation of the co-receptor CD19 activates phosphatidylinositol 3 kinase (PI3K) and its downstream targets including AKT, FOXO and BTK. RAS activation downstream both PLCγ2/PKCβ/RASGRP and GRB2/SOS, promotes the recruitment, dimerization and phosphorylation of RAF, which phosphorylates MEK, which in turn phosphorylates ERK. Activated ERK phosphorylates hundreds of targets in the cytoplasm and nucleus. Active RAS further promotes PI3K signaling through binding to the p110 catalytic subunit and enhancing of its catalytic activity. The main RAS/RAF/MEK/ERK small-molecules inhibitors used to counteract RAS-MAPK signaling in B cell lymphoproliferative disorders are listed next to their main targets. ITAM, immunoreceptor tyrosine-based activation motif; PIP2, phosphatidylinositol-4,5-bisphosphate; PIP3, phosphatidylinositol-3,4,5-triphosphate; IP3, inositol-1, 4,5-triposphate; DAG, diacylglycerol; CBM, CARMA1-BCL10-MALT1; GDP, guanosine diphosphate; GTP, guanosine triphosphate.

Upon antigen binding, LYN or other SRC kinases phosphorylate CD79A/B in their intracellular immunoreceptor tyrosine-based activation motifs (ITAMs). Phosphorylated ITAMs recruit and activate SYK, which in turn activates BTK, PLCγ2, VAV, GRB2, the adaptor protein BLNK, and promote the formation of signalosome. This results in the activation of NF-kB, NFAT and MAPK signaling pathways [5]. In particular, the RAS-MAPK pathway is activated downstream of both PLCγ2/PKCβ/RasGRP and GRB2/SOS and the two activation mechanisms are involved in a positive feedback loop [6]. LYN phosphorylation of the co-receptor CD19 activates PI3K and its downstream targets including AKT and BTK [5]. A scheme of the BCR pathway is depicted in Figure 1.

## 3. KRAS and RAS-MAPK Pathway Aberrations in Mature B Cell Lymphoproliferative Disorders

**Figure 2:**
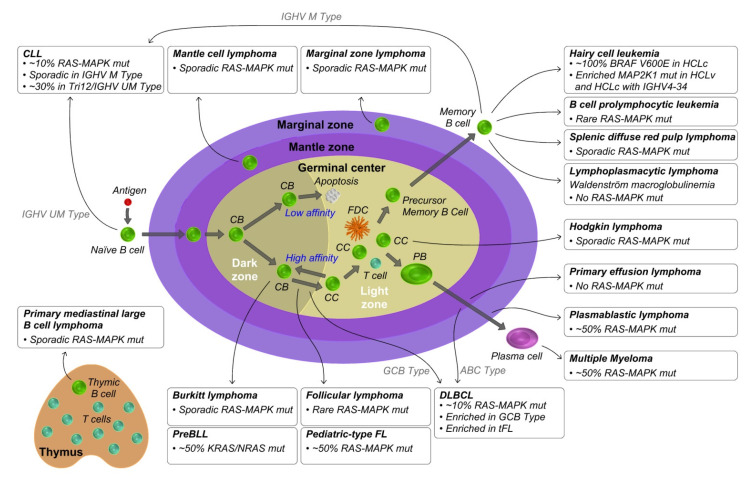
Origin of mature B cell lymphoproliferative disorders and RAS-MAPK pathway deregulation. In the T cell-dependent immune response, the encounter of naïve B cells with the cognate antigens promotes their activation and the formation of germinal centers (GC). In the GC, B cells undergo B cell receptor (BCR) editing through somatic hypermutation (SHM) and class-switch recombination (CSR) to generate B cells with high affinity antibodies of different isotype classes. B cells will differentiate in memory B cells and long-lived plasma cells. In the GC, B cell cycles several times between the dark zone, where proliferation and SHM occur, and the light zone, where interaction with follicular dendritic cells and T cells allow B cell activation and selection on the basis of affinity of their BCR. Acquisition of genetic lesions disrupts normal B cells development and differentiation, leading to malignant transformation. Each mature B cell malignancy is reminiscent of B cells at a particular stage of differentiation, defined as the normal B cell counterpart. The reviewed B cell neoplasms are depicted at the site of their normal counterpart, and incidence of RAS-MAPK aberrations is shown. CB, centroblast; CC, centrocyte; FDC, follicular dendritic cell; PB, plasmablast; mut, mutations; CLL, chronic lymphocytic leukemia; IGHV, immunoglobulin heavy-chain variable region gene; M, mutated; UM, unmutated; Tri12, trisomy 12; HCLc, classic hairy cell leukemia; HCLv, variant hairy cell leukemia; DLBCL, diffuse large B cell lymphoma; GCB, germinal center B cell-like; ABC, activated B cell-like; FL, follicular lymphoma; tFL, DLBCL transformed from follicular lymphoma; PreBLL, precursor B cell phenotype Burkitt leukemia.

Mature B cell neoplasms are a heterogeneous group of hematopoietic disease, presenting with very different morphological, immunophenotypic and clinical features that include B cell prolymphocytic leukemia (B-PLL), Hodgkin’s Lymphoma (HL) and Non-Hodgkin’s Lymphoma (NHL). For each B cell neoplasm it is possible to define a “normal B cell counterpart”, also called a “cell of origin”, on the basis of its phenotypic features reminiscent of B cells at a particular stage of differentiation (Figure 2). Immunoglobulin heavy-chain variable region gene (IGHV) mutational status and gene expression profile of malignant cells mostly take part to the definition of their normal B cell counterpart [7,8].

Virtually all mature B cell lymphoproliferative disorders have been investigated by high-throughput sequencing, and in most of the cases recurrent genetic hallmarks have been identified. Despite this, the depth of genetic knowledge varies by disease, with some malignancies having more than one thousand of cases investigated by WGS or WES, and others limited to less than a dozen cases sequenced, or analyzed primarily by targeted resequencing. Indeed, several aspects of disease presentation might influence the extent of its genomic characterization, including the incidence among the population, the anatomical site of presentation, the portion of non-malignant cells in the tumor milieu, and the degree of genomic instability and complexity. This disparity, which penalizes less frequently mutated genes, will be bridged by the constant improvement in genomic characterization. Grouping genetic aberrations in the main cellular pathways allows us to appreciate the contribution of less frequent mutated genes to the oncogenic process. This is the case of aberrations in the RAS-MAPK pathway, initially identified individually as the hallmark of some lymphoproliferative disorders, that have now emerged as a group of aberrations frequently observed in mature B cell neoplasms.

In this section we review the occurrence and key features of RAS-MAPK aberrations in the context of the main mature B cell neoplasms, focusing on focal genomic aberrations, mostly point mutations, but also small insertions/deletions that represent the genetic events most often involved in RAS-MAPK deregulation. The mutation incidence in each different malignancy is summarized in Figure 2, whereas focal aberrations occurring in the most frequently mutated RAS-MAPK pathway genes, including those encoding for RAS (*KRAS*, *NRAS*, *HRAS*), RAF (*BRAF*, *RAF1*), MEK1/2 (*MAP2K1* and *MAP2K2*), ERK1/2 (*MAPK3* and *MAPK1*) and NF1, are listed in Table 1.

### 3.1. Mantle Cell Lymphoma

Mantle cell lymphoma (MCL) is an uncommon and aggressive NHL that usually affects elderly patients. Nodal MCL (nMCL) is the prevalent MCL subtype, accounting for 80–90% of cases, that mainly presents with the accumulation of malignant cells in the lymph nodes and extranodal sites, and is characterized by unmutated (UM) or minimally mutated IGHV, high expression of the *SOX11* gene, and an aggressive clinical course. The less common MCL form, called non-nodal leukemic MCL (nnMCL), is characterized by leukemic manifestation, mutated IGHV, absent or very low *SOX11* expression and indolent clinical behavior [53]. MCL is historically thought to arise from naïve B cells that reside in the mantle zone of lymphoid follicles [54]; while nMCL maintains the feature of naïve-like mature B cells, nnMCL has features of memory-like B cells and is thought to originate from cells that have passed through the germinal center (GC) [55].

The genetic hallmark of MCL is the chromosomal translocation t(11;14)(q13;q32), leading to high expression of cyclin D1 and constitutive cell cycle deregulation [53]. While nMCL is characterized by very high genomic instability, with numerous structural variants (SV), somatic copy number alterations (SCNA) and recurrent somatic mutations, nnMCL presents with a lower number of genomic alterations and is considered genetically stable [9]. 

RAS-MAPK mutations are uncommon in MCL, with a few *BRAF* or *NRAS* mutated cases found by Nadeu and colleagues [9], one *MAPK3* mutation found in a relapsed MCL case [10] and some *KRAS, BRAF and NF1* mutations identified in a cohort of MCL patients who discontinued ibrutinib treatment [11]. 

### 3.2. Diffuse Large B Cell Lymphoma

Diffuse large B-cell lymphoma (DLBCL) is an aggressive NHL characterized by high genetic, phenotypic and clinical heterogeneity [56]. Based on their gene expression profiles, DLBCLs are divided in activated B cell-like (ABC) and GC B cell-like (GCB) subtypes, while 10–20% of cases remain unclassified [57,58]. ABC DLBCL is thought to derive from antigen-exposed B cells passed through the GC and committed to plasmablastic differentiation [56]. Accordingly, this subtype is characterized by constitutive NF-kB activation; mutations in genes involved in BCR and toll-like receptor signaling and impaired terminal B cell differentiation [59,60,61]. GCB DLBCL is thought to originate from GC light zone B cells [56], frequently presents with activation of PI3K signaling, the alteration of chromatin-modifying enzymes and GC B cell migration, and with the translocation of *BCL2* [62,63,64,65]. The ABC subtype is associated with worse overall survival when compared to GCB DLBCL [14,57].

Multiple mechanisms perturb driver genes, with candidate cancer genes frequently residing in focal SCNA [12]. *IGH*, *BCL2*, *BCL6* and *MYC* are frequently involved in chromosomal rearrangement, and more than 300 recurrently mutated genes have been identified in DLBCL [12,14,66]. 

Mutations in RAS-MAPK genes in DLBCL were firstly observed in the 1990s [67] and then in 2012 with the advent of next generation sequencing [13]. Shortly after, Reddy and colleagues reported the WES of 1001 DLBCL cases and 400 paired germline samples. *KRAS* (*n* = 17), *HRAS* (*n* = 20), *BRAF* (*n* = 19), and *MAP2K1* (*n* = 16) were among the 150 putative driver genes recurrently mutated in DLBCL identified in the study [14]. Overall, 72 cases (~7%) had one RAS-MAPK mutation and no co-occurrence was observed in this dataset. Several *KRAS* and *HRAS* mutations occurring on exon 5 were observed, and no *NRAS* mutations were found in this cohort. Furthermore, *NF1* (*n* = 33) was among the 150 putative driver genes and, in a few cases, *NF1* mutations were co-occurring with *HRAS*, *KRAS* or *MAP2K1* mutations. In this study, mutations of *NF1* and *HRAS* were associated with more favorable prognosis [14]. In a further large study, that involved 574 DLBCLs, an association of *BRAF* (6.1% GCB vs. 1.4% ABC) and *MAP2K1* (6.8% GCB vs. 0.7% ABC) mutations with the GCB subtype was identified. In this study, RAS-MAPK mutations were mostly clonal (defined as >10% allele frequency) [15]. Accordingly, Chapuy and colleagues identified *BRAF* (6% incidence), *KRAS* (3%) and *MAP2K1* (3%) among candidate cancer genes, and mutations in these genes were mostly clonal. By integrating recurrent mutations, SCNAs and SVs, five distinct DLBCL subsets were defined. In subset 4, composed primarily by GCB subtype, *BRAF* alterations were frequently identified and were associated with a favorable outcome [12]. In a study investigating the genomic profile of de novo DLBCL, relapse/refractory (r/r) DLBCL and DLBCL transformed from follicular lymphoma, aberrations of genes affecting the RAS-MAPK pathway were found in 18.4%, 13.8% and 23.1% of cases, respectively [66]. 

Genes in the RAS-MAPK signaling are further involved in amplification (e.g., *BRAF*, *RAF1*, *KRAS*, *HRAS*) or deletions (e.g., *NRAS*, *MAP2K2*) [12]. Moreover, a mutagenesis screening identified the proto-oncogene Ras-responsive element-binding protein 1 (RREB1) as a genetic driver in DLBCL, with an impact on proliferation and KRAS signaling regulation. The study suggests RREB1 upregulation as a mechanism of RAS-MAPK activation in DLBCL [68]. Lastly, *BRAF* and *NRAS* point mutations were identified in primary extranodal DLBCL of the thyroid, suggesting a role of the RAS-MAPK pathway alteration in the pathogenesis of DLBCL developing in the thyroid gland [69,70].

The RAS-MAPK pathway does not come up among the most relevant pathways deregulated in DLBCL, however, the definition of RAS-MAPK mutations still present in a considerable fraction of patients at the clonal level, may be useful in the perspective of targeted therapy.

### 3.3. Follicular Lymphoma

Follicular lymphoma (FL) is the most common and indolent NHL derived from GC B cells. FL usually presents in lymph nodes and ultimately disseminates in the bone marrow (BM) and extranodal sites. Tumor cells grow in GC-like structures in enlarged lymphoid follicles, consist of centroblasts and centrocytes, and the centroblast to centrocyte ratio is informative of the FL pathological grade. FL cells express GC B cell markers, have mutated IGHV genes and resemble normal GC light zone B cells by gene expression profile. The great majority of FL are characterized by the t(14;18)(q32;q21) translocation that results in BCL2 constitutive expression. FL cells go through long preclinical and subclinical phases before overt FL, in which they cycle between GC cell and memory cell states. Multiple GC re-entry cause accumulation of AID-induced genomic aberrations, mainly in histone and chromatin modifying enzymes, that further hijack the GC molecular program with promotion of FL lymphomagenesis and complex subclonal heterogeneity [71,72]. Most patients initially respond to therapy but a large proportion of FL patients experience relapse and a fraction of FL undergoes histological transformation. In most of the cases FL transform to clonally related DLBCL, mainly of the GCB subtype [71]. 

Mutations in the RAS-MAPK pathway genes are a rare event in FL [16,73,74]. However, a high frequency of mutations, including oncogenic hotspots in *KRAS*, *BRAF* and *MAP2K1*, were observed in cases of secondary histiocytic/dendritic neoplasms, developed either concurrently or subsequently to clonally related FL, suggesting a role for RAS-MAPK pathway alteration in the transdifferentiation process [75,76]. Moreover, enrichment of aberrations of genes affecting the RAS-MAPK pathway [66] and NRAS upregulation were observed in FL transformed to DLBCL, supporting a role of RAS-MAPK signaling in FL transformation [77]. 

In the context of an FL variant, the pediatric-type follicular lymphoma (PTFL), more than half of cases present with mutations in RAS-MAPK pathways. PTFL, mostly occurring in pediatric age and in young adults, and only sporadically in older adults, is characterized by localized lymphadenopathy and excellent prognosis. As opposed to typical FL, PTFL lacks *BCL2* gene rearrangements and mutations in epigenetic modifier genes. The most frequently mutated gene is *MAP2K1*, with approximately half of PTFL patients carrying known missense activating mutations, but also *MAPK1* and *RRAS* activating mutations were observed. The high recurrence and mutually exclusivity of mutations in the RAS-MAPK genes suggest a role of RAS-MAPK signaling activation in the unique biology of PTFL [17,78].

### 3.4. Burkitt Lymphoma

Burkitt lymphoma (BL) is an aggressive B cell NHL characterized by fast growing cells and strong invasiveness. BL may present in three different variants, namely the endemic, sporadic, and immunodeficiency-related. The endemic variant is prevalent in young children from malaria-endemic areas of Africa and is associated with Epstein-Barr virus (EBV) infection. Sporadic BL is the predominant variant in non-malarial areas, mostly diagnosed in childhood and less common in adults, and is associated with EBV in 30% of cases. BL of the immunodeficiency-related type is associated with HIV infection or organ transplantation, and 40% of cases present EBV infection [79]. According to its gene expression profile, BL is thought to originate from GC dark zone B cells, even though origin from memory B cells has been proposed for EBV-positive cases [79,80]. *MYC* translocation is the genetic hallmark of BL, generated via aberrant somatic hypermutation or class switch recombination. The translocation is present in 90% of cases, involves the immunoglobulin gene loci (mainly *IGH* but also *IGL* and *IGK*) and results in constitutive MYC activation. 

Genomic aberrations in RAS-MAPK pathway genes are only sporadic in BL, with few missense point mutations identified in *BRAF* [18,19], *MAP2K1* [81], *MAP2K2* [20] and *RAF1* [21]. Instead, genes in the RAS-MAPK pathway are quite often involved in amplification, including *KRAS*, *RAF1* and gain of 7q32-q36 hosting *BRAF* gene [79]. 

Of interest, a rare BL variant characterized by a precursor B cell immunophenotype, called precursor B cell phenotype Burkitt leukemia (preBLL), was shown to present with frequent (50%) *KRAS* (G13D, A146T) and *NRAS* (G13D/R) aberrations [82]. However, it should be noted that several discrepancies with BL, including *MYC* translocations being generated in premature B cells via V(D)J recombination, the frequent lack of functional BCR, and the mutation and DNA methylation patterns closely resembling preB lymphoblastic leukemia/lymphoma (pB-ALL/LBL), call into question the classification of preBLL among BL subtypes [82].

### 3.5. Hodgkin Lymphoma

Hodgkin’s lymphoma (HL) is a mature B cell neoplasm commonly diagnosed in young adults. Classical Hodgkin’s lymphoma (cHL) is the prevalent form and is marked by large mononuclear cells and multinucleate giant cells called Hodgkin and Reed-Sternberg (HRS) cells. HRS cells lack functional BCR and most of the B cell markers, are typically rare (<5% of cells) in the affected lymph nodes, and are embedded in an inflammatory background. Classical HL is further subdivided into nodular sclerosis, mixed cellularity, lymphocyte-depleted and lymphocyte-rich subtypes. The less common form, accounting for 10% of HL, is called nodular lymphocyte-predominant HL (NLPHL) and is characterized by lymphocyte-predominant (LP) cells, also known as lymphocytic and histiocytic cells, that express a GC B cell phenotype. Both HL forms arise from mature B cells; HRS cells are derived from GC B cells that have acquired disadvantageous mutations in the IGHV genes and have failed apoptosis, while LP cells are derived from positively selected GC B cells [83,84].

Sporadic mutations in the RAS-MAPK pathway genes were found, including *NRAS*, *BRAF*, *MAP2K1* and *NF1* [22,23,85,86]. A single case with *KRAS* L19F mutation was reported in the literature [24]. In a cHL case, *KRAS* mutation (G60D) was found in cells from clonal hematopoiesis but not in HRS cells; indeed, clonal hematopoiesis of indeterminate potential was shown to occur in a fraction (12.5%) of cHL cases, including in young patients [87].

### 3.6. Primary Mediastinal Large B Cell Lymphoma

Primary mediastinal large B-cell lymphoma (PMBL) is a rare aggressive B cell NHL mostly diagnosed in young women and typically presenting with large mediastinal mass. PMBL cells present as large cells expressing typical B cell antigens but lacking surface immunoglobulins. PMBLs are thought to arise in the thymus from transformed thymic medullary B cells [88]. While sharing morphological features with DLBCL, PMBL transcriptional and molecular features are closely related to cHL [88]. 

Only sporadic mutations in the RAS-MAPK genes were identified [89,90].

### 3.7. Chronic Lymphocytic Leukemia

Chronic lymphocytic leukemia (CLL) is an indolent mature B cell neoplasm representing the most common leukemia in the Western world. CLL is characterized by the accumulation of mature clonal B lymphocytes in the blood, BM and other secondary lymphoid tissues. CLL is characterized by remarkable clinical heterogeneity, ranging from an indolent disease with no requirement for treatment in some patients to rapid disease progression and subsequent treatment refractoriness in others [91]. CLL may undergo histologic transformation into an aggressive B cell lymphoma, commonly DLBCL or HL, a process termed Richter’s transformation (RT), associated with a very dismal clinical outcome [92]. A plethora of prognostic factors, both clinical and biological parameters, has been identified to allow a better prediction of the individual prognosis of a given patient, including disease stage, the presence of chromosomal abnormalities (see below), IGHV mutational status, gene mutations (such as *TP53*, *NOTCH1*, *SF3B1*, *BIRC3*), and surface antigen expression (CD49d, CD38, ZAP-70) [93,94,95]. While the chromosomal abnormalities trisomy 12 (tri12), 13q14 deletion (del13) and 11q22 deletion (del11) have been suggested as early driver events in CLL onset observed to remain stably clonal during disease evolution, the deletion of 17p13 (del17) and recurrent gene mutations are considered late driver events [25]. Most frequently mutated genes include *NOTCH1*, *TP53*, *ATM*, *SF3B1*, *BIRC3*, *CHD2*, *MYD88*, *POT1* and *XPO1* [25,26,31].

Early observation of RAS mutations in CLL date back to the 1990s [67]. More recently, in a large WES study investigating a CLL cohort of 538 cases, Landau and colleagues revealed an 8.7% mutation incidence in RAS-MAPK pathway genes, including *KRAS* and *NRAS* (4.1%), *BRAF* (3.7%) and *MAP2K1* (2%). Mutations were mostly subclonal and defined late driver events in CLL [25]. A comparable mutation incidence in these genes was observed in other studies [26,27,28,96]. With a lower frequency, mutations were found in additional genes of the RAS-MAPK pathway (*MAPK1*, *MAP2K2*, *RAF1* and *NF1*) or coding for upstream regulatory proteins, such as *KITLG*, *KIT*, *PTPN11*, *GNB1* [29]. *KRAS* and *NRAS* mutations largely occur in the hotspots codons G12, G13, Q61 and A146, while *BRAF* mutations only rarely involve the canonical V600E mutation and mainly occur nearby, in the activation segment of kinase domain [25,26,30,31]. Of note, *BRAF* V600E mutation was found to be enriched in a cohort of patients that underwent RT [97]. *MAP2K1* mutations were enriched in previously treated patients, suggesting that these mutations might be selected by the therapy [25].

RAS-MAPK mutations were mostly associated to IGHV UM, tri12 and tri12-associated features like high expression of ZAP-70, CD49d and CD38 [27,29]. Of note, the *KRAS* gene, one of the more frequently mutated genes in the pathway, is located on chromosome 12. Furthermore, RAS-MAPK mutations were associated with worse treatment free survival (TFS) [29]. In a CLL cohort, purposely enriched in IGHV UM and tri12 CLL cases we showed that up to 90% of *RAS*/*RAF* aberrations occur in IGHV UM CLL, and up to 80% occur in cases bearing tri12 [30]. In particular, *RAS*/*RAF* mutations were found in roughly 30% of patients with IGHV UM and tri12 as sole chromosomal aberration (tri12-only, without co-occurring del13q, del11q or del17p), gaining a place among the most frequently mutated genes in the group alongside *NOTCH1* [98,99]. Of note, *RAS*/*RAF* mutation frequency was much lower in other cytogenetic defined groups, with up to 16% incidence found among IGHV UM patients bearing tri12 plus other cytogenetic aberrations, mostly del13q but also del11q and del17p, and only 4% incidence detected in patients with del13q as sole chromosomal aberration, highlighting how the type of genomic structural variants strongly influenced *RAS*/*RAF* mutation incidence in CLL. A tendency toward mutually exclusivity of *RAS*/*RAF* mutations with *NOTCH1* and *BIRC3* aberrations was noted. In the context of the tri12/IGHV UM group, the association of *RAS*/*RAF* mutations with high expression of ZAP-70, CD49d, CD38 was lost. Indeed, the association of *RAS* mutations with a shorter TFS was retained in the whole cohort and in the contest of the most heavily mutated group, defined as tri12-only/IGHV UM/*NOTCH1* UM [30]. *BRAF* mutations showed no prognostic value in some studies [25,30], whereas they were associated to shorter TFS or overall survival as independent prognostic drivers in some other studies [26,31,96]

Impaired response to therapy and drug resistance were frequently observed in RAS-MAPK mutated CLL; in particular, *KRAS* mutations were associated with worse overall response to lenalidomide-based therapy [28], and with no response to chlorambucil/CD20-antibody chemoimmunotherapy [27]. *BRAF* mutations were associated with acquired resistance to the BCL2 inhibitor venetoclax [100] and with fludarabine refractoriness [101]. 

Gene expression analysis of CLL cases carrying RAS-MAPK mutations revealed the upregulation of genes of the MAPK pathway. Accordingly, high levels of endogenous phospho ERK was found in mutated CLL, proving activation of the RAS-MAPK pathway in the group [29]. Enrichment of *KRAS*, *BRAF* and *MAP2K1* activating mutations were observed in CLL patients who did not respond to PI3K inhibitors, and persistent ERK activation was shown to mediate PI3K inhibitor resistance in CLL [102]. In this study, combination of MEK inhibitors (CI-1040 and trametinib) with the PI3K inhibitor idelalisib proved to overcome PI3K inhibitor resistance in vitro [102]. 

### 3.8. Hairy Cell Leukemia 

Hairy cell leukemia (HCL) is a chronic lymphoproliferative disorder characterized by peculiar leukemic cells with abundant cytoplasm and hairy-looking projections. HCL cells mostly present features of post-GC memory B cells, with expression of CD19, surface immunoglobulin and with clonal rearrangements of immunoglobulin genes. HCL cells are typically found in the BM, spleen, liver and, in lower amounts, in the blood [103]. Classic HCL (HCLc) is characterized by the expression of CD11c, CD103, CD123 and CD25 in the surface of leukemic cells, the indolent course of disease and good response to purine nucleoside analog therapy. Ten percent of patients lack the specific surface immunophenotype and present a more aggressive disease with worse response to standard therapies; this is known as the HCL variant (HCLv) [104].

Near all HCLc patients harbour the *BRAF* V600E mutation [32]. The *BRAF* V600E mutation is a disease-defining genetic aberration, shown to be somatic, clonal and stable during the course of disease. *BRAF* V600E mutations are mostly heterozygous, with the exception of patients in which co-occurring 7q deletion causes the loss of the wild-type allele [103]. The *BRAF* V600E mutation, which occurs on exon 15, constitutively activates the BRAF kinase activity and the downstream MEK/ERK signaling pathway [105], primarily contributing to HCL pathobiology. Sustained MEK/ERK signaling promotes HCL cells survival [106], but more than this, BRAF V600E signaling shapes all main tracts of HCL biological identity, as shown in the BRAF inhibition experiments. Indeed, the use of BRAF inhibitors in vitro determines the loss of typical gene expression, immunophenotype and morphologic characteristics of hairy cells, with induction of apoptosis [107]. *BRAF* V600E mutations are absent in the subgroup of HCLc cases with IGHV4-34 rearrangement and in HCLv cases [108]. Patients in these two groups are enriched with mutations in the *MAP2K1* gene that result in MEK/ERK pathway activation. Unfortunately, the majority of these *MAP2K1* mutations make cells insensitive to current MEK inhibitors due to the disruption of the inhibitor binding domain [33]. Alternative mutations in exon 11 of the *BRAF* gene, namely F468C and D449E, were identified in two HCL cases without the canonical V600E aberration, and in a single case with *BRAF* V600E, a second *BRAF* exon 15 aberration was identified (namely S602T) [35]. 

Although many HCL cases have been screened for the presence or absence of the *BRAF* V600E point mutation, most frequently the assays design strictly included the hotspot V600 codon, and the *BRAF* exon 15 and exon 11 were sequenced at a deep level in only a minority of cases. In sharp contrast with other NHL subtypes, less than 20 HCL cases have been analyzed by WES in the reviewed literature [32,33,109,110], and in one study a large panel of tumour-related genes has been investigated by target sequencing in a relatively large cohort (61 HCL patients) [34]. In this latter study, *KRAS* and *NRAS* activating mutations were identified. Since these mutations are known to cooperate with the BRAF inhibitor-mediated paradoxical ERK activation and resistance mechanisms, assessing their presence may be of value for therapeutic choices. 

### 3.9. Other Post Germinal Center Lymphomas

Marginal zone lymphomas (MZL) originate from the marginal zone B cells and may involve the spleen, defined splenic MZL (SMZL), the lymph nodes, defined nodal MZL (NMZL), or the extranodal sites, defined extranodal marginal zone lymphoma (EMZL) of mucosa-associated lymphoid tissue (MALT). SMZL and NMZL are characterized by aberrations of the *NOTCH2*, *KLF2* and NF-kB signaling genes involved in the commitment of mature B cells to the marginal zone [111]. Furthermore, NMZL is associated with recurrent molecular lesions of *PTPRD* [112]. Sporadic somatic point mutations of RAS-MAPK genes were identified in SMZL, including *KRAS*, *BRAF*, *RAF1*, *MAP2K1*, *MAP2K2* and *NF1,* as reported in a systematic review including WES and targeted resequencing studies [39]. In NMZL only *BRAF* mutations, including V600E, have been reported so far [40]. 

MALT lymphoma occurs at diverse anatomic sites and is associated with chronic inflammatory disorders, such as chronic infections and autoimmune disorders. MALT lymphoma originates from the marginal zone B cells of the acquired MALT, and the pathogenesis is centered on activation of the NF-kB pathway triggered by genetic changes and immunological stimulation, including chronic BCR activation by infectious agents like *Helicobacter pylori* or autoantigens [113]. Recurrent chromosomal translocations t(11;18), t(14;18), and t(1;14) are detected in MALT lymphomas, affecting *BIRC3*/*MALT1*, *MALT1*, and *BCL10* genes, respectively. Deregulation of the RAS-MAPK pathway seems not to be involved in the disease, and RAS-MAPK aberrations are only sporadically reported [41,42]. 

Splenic diffuse red pulp lymphoma (SDRPL) is a rare small B cell lymphoma characterized by villous lymphocytes circulating in the peripheral blood, BM and spleen. SDRPL presents similarity with SMZL, HCL and HCLv. In the literature, only a single study presented deep sequencing data from SDRPL cases, highlighting the recurrence of BCL6 corepressor (BCOR) aberrations in these cases [38]. The study further revealed sporadic mutations targeting several genes in the RAS-MAPK pathway [38]. Similarly, two *MAP2K1* mutated cases and one *BRAF* mutated case were previously identified in a cohort of 19 SDRPL patients analyzed by Sanger sequencing [43], confirming that RAS-MAPK mutations, although infrequent, are part of the genomic landscape of SDRPL.

B cell prolymphocytic leukemia (B-PLL) is a rare mature B cell malignancy, characterized by the accumulation of clonal prolymphocytes in the peripheral blood. B-PLL mostly presents as aggressive disease with poor prognosis, and mostly affects elderly patients. B-PLL closely resembles CLL and MCL; the leukemic cells have clonal IGHV gene rearrangements and present with both mutated or UM IGHV genes. B-PLL is characterized by a complex karyotype, including trisomy 12, and frequent aberrations of *TP53* and *MYC* [114,115]. The rarity of the disease prevented genomic characterization in large series, and a study reporting WES analysis on 16 B-PLL cases revealed the absence of recurrent RAS-MAPK mutations [116]. Only one case of B-PLL with a *BRAF* V600E mutation in heterozygosis was reported to date, associated to trisomy 12 [44].

Waldenström macroglobulinemia (WM) is the most frequent form of lymphoplasmacytic lymphoma (LPL), and is characterized by the accumulation, in the BM, lymph nodes, and spleen, of clonally related lymphocytes, lymphoplasmacytic cells and plasma cells, which secrete monoclonal IgM proteins. Less frequent forms of LPL include IgA secreting, IgG secreting and nonsecreting LPL [117,118]. WM cells generally express post-GC features and likely originate from memory B cells that have experienced GC reaction and retain the capacity to undergo plasma cell differentiation [119]. No mutations in the RAS-MAPK pathway were reported in WM [120,121,122] though limited WGS, and WES data are available in the literature. 

### 3.10. Multiple Myeloma 

Monoclonal expansion of plasma cells in the BM and the abnormal production of immunoglobulins, called M proteins, characterize multiple myeloma (MM), also known as plasma cell *myeloma*. MM progresses from the early pre-malignant stage defined by monoclonal gammopathy of undetermined significance (MGUS) to the asymptomatic smoldering MM (SMM), to the overt symptomatic MM, until reaching the most aggressive stage characterized by plasma cell leukemia (PCL) and extra medullary disease. Primary cytogenetic aberrations are thought to be an early event in the transformation from polyclonal to monoclonal plasma cells. The acquisition of trisomies of odd-numbered chromosomes (3, 5, 7, 9, 11, 15, 19 and 21) characterizes the hyperdiploid MM subtype, whereas translocations of the IGH locus at chromosome 14q32 with various partner chromosomes (most frequently 4, 6, 11, 16, and 20) characterizes the nonhyperdiploid subtype [123]. MM is characterized by extreme genetic heterogeneity both among patients, presenting their own composite of chromosomal rearrangements and gene mutations, and intraclonally, with most of the patients presenting a complex subclonal structure [46].

The identification of *KRAS* and *NRAS* mutations in MM dates back to the 1980 and was the first description of the involvement of RAS aberrations in mature B cell malignancies [2].

To date, with more than one thousand MM cases sequenced by WGS or WES, the RAS-MAPK pathway appears to be the most frequently mutated pathway in MM. With minor variation among the different studies, the reported mutation incidence is 22–25% for *KRAS*, 20–25% for *NRAS* and 6–15% for *BRAF,* with up to 50% of newly diagnosed MM cases affected by RAS-MAPK pathway aberration on the whole [45,46,47,124]. The most frequent hotspot mutations are codons 12, 13 and 61 in the *KRAS* and *NRAS* genes, with a prevalence of Q61 mutations for *NRAS* [125], and codon V600 in the *BRAF* gene. *KRAS*, *NRAS* and *BRAF* mutations were frequently identified in high cancer cell fractions [47] and considered driver mutations in MM. Mutations were found to be both clonal in some patients and subclonal in others, suggesting that even driver mutations can be acquired late in the disease progression [46]. A study on an MM cohort with long-term follow-up data revealed *BRAF* mutations being associated with an adverse outcome. While the most frequent *BRAF* V600E mutation results in constitutive BRAF activation, a substantial part of non-V600E *BRAF* mutations consists of hypoactive or kinase-dead mutations. Notably, patients with inactivating *BRAF* mutations had worse outcomes than those with *BRAF* activating mutations [126]. 

The large amount of genomic data available for newly diagnosed MM, allowed the identification of the association and mutual exclusion between driver aberrations. *KRAS* mutations were found to positively correlate with t(11;14) [47], whereas *NRAS* mutations negatively correlate with t(4;14) [124,125]. *BRAF* mutations are enriched in t(14;16) group where a prevalence of mutations in the codon D594 is observed [125]. Less frequently, mutations are found in other genes part of the RAS-MAPK signaling, like *NF1* and *RASA2*, both negative regulators of RAS proteins, and *PTPN11*, *PRKD2, FGFR3*, positive RAS regulators [125]. *FGFR3* and *PRKD2* aberrations were associated to t(4;14), whereas *PTPN11* and *RASA2* aberrations were associated to hyperdiploidy [125]. Furthermore, fusion genes involving *BRAF*, *NTRK3*, *ALK*, *FGFR1*, and *ROS1*, shown to activate MEK/ERK pathway in other cancers, were described [127]. 

Activation of the MEK/ERK pathway mediates MM cell proliferation, survival and migration [128], and accumulation of RAS/RAF pathway aberrations is deeply involved in the progression, relapse and drug resistance in MM. Indeed, the frequency of *KRAS*, *NRAS* and *BRAF* mutations increases from 24% in SMM [129], to 50% in newly diagnosed MM, and up to 72% among MM cases refractory to proteasome inhibitors (PIs) and/or immunomodulatory drugs (IMiDs) [130]. 

*KRAS* mutations have a critical role in MGUS to MM progression, with *KRAS* mutations rarely identified in MGUS cases [131] but being the most frequently mutated gene in SMM, and shown to be independently associated with a shorter time to progression from SMM to MM [129]. Likewise, *NRAS* mutations are less frequent in SMM when compared to MM, consistent with a role as drivers of progression [129].

*NRAS* mutation seems to be crucial in the promotion of drug resistance, indeed a prevalence of *NRAS* mutations are observed in r/r MM [48]. Accordingly, *NRAS* mutations are associated with reduced sensitivity to PI bortezomib in relapsed MM [132]. 

### 3.11. Other Plasma Cell Related Diseases 

Plasmablastic lymphoma (PBL) is an aggressive B cell lymphoma with poor prognosis, characterized by large neoplastic cells with plasmablast morphology and plasma cell phenotype. PBL mostly occurs in adult patients with HIV infection or with iatrogenic immunodeficiency, and predominantly have extranodal presentation. It is considered a rare disease, but cases increase dramatically in the world regions with high HIV incidence [49]. PBL is supposed to derive from post-GC B cells in the transition toward plasma cell differentiation. PBL cells are characterized by the downregulation of B cell antigens and BCR signaling genes, and by the high expression of plasma cell markers. PBL is frequently associated with EBV infection and *MYC* rearrangements. *MYC* mutations that can be ascribed to an aberrant somatic hypermutation pattern were also reported [49].

The mutational profile of PBL appeared only very recently in the literature, and revealed up to 49% of cases carrying RAS-MAPK pathway aberrations [49,50,51,52]. *NRAS* gene mutations were prevalent, detected in up to 33% of cases and mostly occurring in G12, G13 and Q61 hotspots. *KRAS* mutations were found in up to 12% of cases, *BRAF* and *MAP2K1* gene mutations each occurred in up to 7% of PBL cases, and *HRAS* had 2% frequency. Occasionally, mutations in other RAS-MAPK pathway related genes like *RAF1*, *MAPK1*, *MAPK3* or *NF1* were found.

Co-occurrence of mutations in multiple RAS-MAPK genes were observed, with the exception of *KRAS* and *NRAS* mutations that were mutually exclusive. Most of the mutations lead to constitutive activation of the RAS-MAPK pathway, and despite the identification of subclonal aberrations, most of the RAS-MAPK mutations were clonal and probably drivers in the oncogenic process [52]. In the cases investigated by Liu et al., *RAS*/*RAF* mutations were found in heterozygosis and mutant alleles were actively expressed [49]. Genes in the RAS-MAPK pathway are further involved in chromosome gain or amplification [49,50,51] in PBL, and recurrent amplification of the 8q24.13 region was reported, encompassing the *TRIB1* gene, which participates in MEK/ERK signaling activation [52]. Specific associations or mutual exclusions of RAS-MAPK mutations with other molecular events are not highlighted in the current literature but could be of interest, especially in the context of the NOTCH1 pathway aberrations recurrently observed in PBL [49]. In regard to the association with prognosis, Frontzek et al. reported a trend toward unfavorable outcomes in PBL patients with *NRAS* aberrations [52]. Recurrent *RAS*/*RAF* aberrations (55% of mutation incidence) were also identified in post-transplant PBLs, a rare type of post-transplant lymphoproliferative disorder accounting for 5–14% of PBL cases [133]. The ctivation of RAS-MAPK signaling emerges as a pathogenetic mechanism in plasma cell dyscrasias, [49] and the therapeutic potential of RAS/RAF/MEK/ERK inhibition should be addressed in PBL [52]. 

Primary effusion lymphoma (PEL) is a rare and aggressive B cell lymphoma that commonly presents as malignant effusions in the body cavities without detectable tumor masses. A rare subtype of this disease, called extracavitary PEL, presents with solid tumor mass in extranodal sites. PEL mostly affects immunocompromised patients, prevalently with HIV infection, or elderly patients, and is generally associated with poor prognosis [134]. PEL is thought to originate from post-GC B cells with plasmablastic differentiation, malignant cells usually lack B cell markers and immunoglobulin expression, while hold markers of plasma cell differentiation

Very little is known about the genomic landscape of PEL, mostly due to the rarity of the disease. PEL is characterized by complex karyotypes with many chromosomal aberrations. Gaidano et al. observed the frequent occurrence of complete or partial trisomy of chromosome 12 and chromosome 7, hosting *KRAS* and *BRAF*, respectively, in PEL cell lines [135]. Furthermore, amplification of *RAF1* was reported in EBV negative PEL cell lines [136]. WES of PEL cell lines revealed mutations mostly ascribed to the APOBEC mutational signature, and no mutations in the RAS-MAPK pathway genes were reported [137]. Similarly, in an additional target deep sequencing analysis of 12 primary PEL cases, no mutations were found in the *BRAF* gene, the only member of the RAS-MAPK pathway included in the 36-genes lymphopanel investigated [138].

## 4. RAS-MAPK Pathway Inhibitors 

The hyperactivation of RAS-MAPK signaling has long been regarded as a key therapeutic target in cancer. First-generation RAF inhibitors (such as vemurafenib, dabrafenib, encorafenib), selective for the BRAF V600E mutant, showed efficacy in BRAF V600E mutated cancers. Unfortunately, resistance mechanisms inevitably emerged, mostly based on ERK pathway reactivation. New-generation RAF inhibitors (such as TAK-580), and numerous RAS, MEK, and ERK small molecule inhibitors allow the targeting of the RAS-MAPK pathway at different levels, as shown in Figure 1. Their use as single agents or in combination, such as dual RAF/MEK inhibition, is giving promising results [3]. Recurrent aberrant activation of RAS-MAPK signaling in mature B cell neoplasms, frequently associated with disease progression and drug resistance, has encouraged exploration of the efficacy of RAS/RAF/MEK/ERK inhibitors in many of these diseases. In this section we outline the main strategies to counteract the aberrant RAS-MAPK signaling that have been evaluated in the different malignancies of the mature B cell. The subclonal nature of leukemias and lymphomas adds a degree of difficulty to the targeting of RAS-MAPK, with single-agent therapy often promoting the emergence of resistant subclones. Several combinatorial therapeutic strategies are currently under evaluation. Reviewed clinical and pre-clinical therapeutic approaches are summarized in Table 2.

### 4.1. RAF Inhibitors

#### 4.1.1. Clinical Setting

Currently, three BRAF inhibitors are approved in Europe and US for the treatment of patients with *BRAF*-mutant cancers, namely vemurafenib, dabrafenib and encorafenib. These drugs are orally bioavailable, ATP-competitive, small-molecule inhibitors of BRAF V600E kinase [186]. 

Early case reports on the use of the BRAF inhibitor vemurafenib in MM patients with the *BRAF* V600E mutation has shown some clinical activity [145,146]. In particular, a case report on the use of vemurafenib in a MM patient with *BRAF* V600E mutation and the absence of *RAS* aberrations showed durable response with no signs of progressive disease or secondary malignancies after eight months [143]. Follow up of the same case was lately reported showing the acquisition of *NRAS* mutations. In this patient, the use of vemurafenib in combination with PI bortezomib proved to be efficacious [144]. The rationale for using the PI and RAF/MEK inhibitor combinations in patients with RAS-MAPK pathway constitutive activation come from two relevant observations. One is that PI bortezomib has been shown to counteract RAS-MAPK signaling while decreasing ERK phosphorylation levels [187]. In addition, activating MAPK pathway mutations increases proteasome capacity while boosting PI resistance. Pharmacologic RAF or MEK inhibition was shown to decrease proteasome activity and sensitized myeloma cells to PIs. Accordingly, in an MM in vivo murine model carrying an Nras activating mutation, the PI/MEK inhibitor combination showed enhanced activity [174].

Besides these initial reports, the use of vemurafenib in clinical trials had only a limited effect among MM *BRAF* V600E mutated patients. In the phase II NCT01524978 basket trial, three out of five MM patients initially enrolled reached disease stabilization [141]. A subsequent report on the same study revealed that two out of nine MM patients had encouraging and long-lasting responses to treatment, and one additional patient had a shorter response [142]. This latter patient, who relapsed after achieving a partial remission, had acquired an *NRAS* mutation and was switched to a clinical trial that test the combination of BRAF inhibitor dabrafenib and MEK inhibitor trametinib (NCT03091257).

In patients with HCL, the use of vemurafenib induces complete or partial response in 96–100% of cases [139]. Unfortunately, all patients retain residual disease and persistence of ERK phosphorylation at the end of treatment with both vemurafenib or dabrafenib, and the vast majority experience relapse [139,151,188]. 

The mechanisms of resistance to BRAF inhibitor therapy in HCL are associated with the acquisition of aberrations within the MEK/ERK signaling pathway (*KRAS*, *MAP2K1* and *IRS1* activating mutations or *NF1* and *NF2* deletions) that reactivate the pathway despite BRAF blockade [34,139,147]. Furthermore, a case of acute myeloid lymphoma (AML) development during vemurafenib treatment was reported, and a PI3K E545K activating mutation was identified in the AML clone, suggesting a potential additional mechanism of MAPK pathway reactivation [188]. Currently, the use of BRAF/MEK inhibitors (dabrafenib plus trametinib) are being evaluated in clinical trial in r/r HCL patients, with promising results [152]. An impressive response was obtained from the combination of vemurafenib plus the anti-CD20 monoclonal antibody rituximab that showed a durable complete response in most patients with r/r HCL [150].

Sorafenib, an RAF and multi-target kinase inhibitor, has shown a very limited activity as a single agent in unselected patients with r/r MM, leading to stable disease for several months in a minority of patients [154,155]. Similarly, the evaluation of sorafenib as a single agent in patients with r/r DLBCL revealed acceptable tolerability but very low activity [160,161]. In CLL, two phase II clinical trials evaluating sorafenib in relapsed patients (NCT00303966, NCT01510756) have been terminated early with only very few recruited patients (five and four, respectively) and no data available in the literature.

In a phase II clinical trial, the combination of sorafenib and the AKT inhibitor perifosine induced a noteworthy response rate in cHL patients, though the disease regression was not durable in the majority of these patients, and induced a partial response in one out of four CLL patients included in the study [169].

#### 4.1.2. Preclinical Studies

Sorafenib was also investigated in several preclinical studies. The combination of sorafenib with MEK inhibitors synergistically potentiated apoptosis in DLBCL cells [162]. In the context of cHL, sorafenib combined with the HDAC inhibitor Givinostat, synergistically inhibited growth, and induced necroptosis in the cHL cell line and xenograft model [167]. In MCL cell lines and primary samples, sorafenib induced apoptosis and impairment of BCR signaling, showing a strong synergism with the SYK inhibitor R406. Moreover, sorafenib modulated cells response to signals from the microenvironment, overcoming stroma-mediated resistance to bortezomib. Accordingly, the combination of sorafenib plus bortezomib synergistically reduced tumor growth in an in vivo xenograft MCL model [165].

In another preclinical study using the FL, DLBCL and BL cell lines, sorafenib induced apoptosis and inhibition of proliferation and had a synergistic cytotoxic effect in combination with the mTOR inhibitor rapamycin [166].

Other strategies demonstrated to be effective in preclinical models. AZD4785, an antisense oligonucleotide which selectively targets and downregulates all KRAS isoforms, has shown good preclinical efficacy both in in vitro and in vivo models of *KRAS* mutated MM, both alone or in a combinatory regimen with the PI bortezomib [170]. TAK-580, a novel pan-RAF inhibitor that acts by disrupting RAF homo- or heterodimerization, has shown response either alone or in combination with PIs and IMiDs in MM cell lines [153].

### 4.2. MEK Inhibitors

#### 4.2.1. Clinical Setting

Since MAPK-pathway reactivation is a frequent resistance mechanism in BRAF inhibitor monotherapy, combined BRAF and MEK inhibition strategies have been developed. Currently, three MEK inhibitors, namely binimetinib, cobimetinib and trametinib, are approved in Europe and US for the treatment of cancer patients [186].

The combined BRAF and MEK blockade has been evaluated in MM cases, and in the first case report on the combination of vemurafenib and cobimetinib, a patient, with highly resistant MM and harboring the *BRAF* V600E mutation, achieved rapid and complete response [148]. Similarly, another case report described prolonged progression free survival in a MM patients, with *BRAF* V600E mutation and disease progression despite multiple lines of therapy, treated with the vemurafenib/cobimetinib combination [149]. Two clinical trials are currently addressing the effectiveness of combined BRAF/MEK inhibition in r/r MM, evaluating encorafenib plus binimetinib (NCT02834364) and dabrafenib plus trametinib (NCT03091257).

Furthermore, the opportunity to target RAF and MEK with a single drug has been explored. Treatment of a subgroup of heavily pretreated MM patients with CH5126766, a dual RAF-MEK inhibitor, induced durable partial response in one patient and durable disease stabilization in another, both patients carrying *KRAS* mutations [171].

MEK inhibitors used as single-agents were also evaluated in MM. The MEK inhibitor selumetinib (AZD6244) resulted in only minimal responses in r/r MM patients, and the response rate was not linked to the *RAS*/*RAF* mutational status [179]. Also in r/r DLBCL patients, the use of the single agent selumetinib demonstrated low tolerability and very limited efficacy [180].

Conversely, in a retrospective study, the MEK inhibitor trametinib showed a 40% response rate among 40 patients with MAPK pathway-activated MM [173]. A preliminary report on the MEK inhibitor cobimetinib, evaluated in the clinical trial NCT03312530, showed no response when used as single agent in MM. Moderate activity was described for the combinations of cobimetinib with the BCL2 inhibitor venetoclax, and particular high activity was shown in t(11;14) MM patients [176].

#### 4.2.2. Preclinical Studies

Very interesting results were obtained from the use of selumetinib in combination with pan-HDAC or class I HDAC inhibitors in MM cells. Indeed, combining selumetinib with LBH589 (panobinostat) or FK228 (romidepsin) induced synergistic apoptosis in *RAS*/*RAF* mutated MM cell lines [182].

In DLBCL cell line models, a synergistic effect was observed for the MEK inhibitor trametinib and the BTK inhibitor tirabrutinib [175]. Similarly, strong synergism was observed for the MEK inhibitor pimasertib combined with the PI3K-delta inhibitor idelalisib or with the BTK inhibitor ibrutinib in DLBCL (particularly the ABC type), in MCL cell lines, and in an ABC DLBCL in vivo xenograft model [183]. 

In CLL, an in vitro drugs screening revealed increased sensitivity to MEK (selumetinib, cobimetinib, trametinib) and ERK (SCH772984) inhibitors among tri12 and *KRAS* mutated cells [189]. In another study, the MEK inhibitor binimetinib reduced cell proliferation and survival in CLL cells under conditions that mimic the tumour microenvironment, and sensitized CLL cells to the BH3-mimetics ABT-737 and venetoclax [177]. Furthermore, a synergistic effect in reducing CLL cells survival and proliferation was reported for binimetinib in combination with the AKT inhibitor MK2206 [178]. The rationale for dual inhibition of the RAS-MAPK and PI3K/AKT/mTOR pathways arise from the recurrent observation that the inhibition of only one of the pathways can result in the in paradoxical activation of the other pathway [178].

Similarly, in cHL, the complex cross-talks between PI3K/AKT and MAPK signaling, both active in malignant cells, prompted the use of the PI3K/ERK dual inhibitor AEZS-136 and demonstrated efficacy in impairing cell proliferation and inducing necroptosis in cHL cell lines and xenograft models [185]. 

In MCL, the opportunity to block the RAS-MAPK pathway downstream to ERK was investigated. RSK2, a kinase downstream to RAS-MAPK signaling, was frequently found to be constitutively active in the MCL cell line and primary samples, independently from RAS/MEK/ERK activation. BI-D1870, a specific inhibitor of RSK2 N-terminal kinase domain (NTKD), induced growth inhibition and apoptosis in MCL lines, suggesting RSK2 NTKD as a potential therapeutic target in MCL [190].

## 5. Oncogenic RAS/RAF Mouse Model 

Genetically engineered mouse models are precious tools for the comprehension of cancer biology, that allow to evaluate tumor interactions with the microenvironment, to understand the relevance of specific genetic aberrations, and to test the efficacy of therapeutic agents. In this section we present some examples of constitutive or conditional mouse models recapitulating genetic lesions in the RAS-MAPK pathway genes frequently observed in mature B cell malignancies. The conditional expression of an oncogenic version of *Ras*/*Raf* genes at specific developmental stages or in specific hematopoietic compartments adds to their role in the development and progression of cancers, allowing for the definition of early and late events in disease pathogenesis. Reviewed mouse models are summarized in Table 3. 

### 5.1. KRAS

The expression of oncogenic Kras G12D has been extensively modeled in the different hematopoietic compartments of transgenic mice. Conditional Kras G12D expression in hematopoietic stem cell (HSC), under the control of *Mx1-Cre*, lead to myeloproliferative disease (MPD) reminiscent of chronic myelomonocytic leukemia (CMML) or juvenile myelomonocytic leukemia (JMML) in all mice, and to concomitant T cell leukemia in a small fraction of mice. Expression restricted to BM compartment, upon BM transplantation in recipient mice, lead to aggressive T acute lymphoblastic leukemia (ALL) enriched with *Notch1* mutations [192].

Conditional Kras G12D expression in post-GC B cells (*Cγ1-Cre*) and in B cells undergoing GC reaction (*AID-Cre*) failed to induce MM or other B cell malignancies, and only minimal B cells perturbation was induced when Kras G12D was expressed in the context of Arf pathway inactivation, a tumor-prone condition known to cooperate with Kras mutations [193].

Conditional Kras A146T expression in HSC (*Mx1-Cre*) lead to myelodysplastic syndrome/myeloproliferative neoplasm (MDS/MPN) with the expansion of immature myeloid cells in the BM and spleen. The onset of disease was delayed and the death occurred at an older age in comparison to Kras G12D mice, in line with a weak tumorigenic potential of the A146T mutant form [194]. Constitutive expression of Kras V14I mutation that recapitulates the most phenotypic feature of Noonan syndrome leads to the development of MPD reminiscent of human JMML. Transplantation of BM cells from Kras V14I/+ mice leads to the development of MPD in recipient mice [195].

### 5.2. NRAS

Similarly to Kras G12D, Nras G12D has been extensively investigated in mouse models. In particular, Wang and colleagues investigated the effect of oncogenic Nras G12D signaling in different cellular contexts and in a gradient of expression levels [196]. When expressed at early embryonic stage, Nras G12D/+ was sufficient to cause embryonic lethality, whereas expression below the endogenous level did not cause abnormalities and cancers. When expressed in post-natal hematopoietic cells under the control of *Mx1-Cre*, Nras G12D/+ leads to either histiocytic sarcoma or a chronic MPD resembling CMML in 50% of mice, whereas Nras G12D/G12D leads to acute MPD with the expansion of myeloid compartments in all mice. When expression was restricted to the BM compartment, upon BM transplantation in recipient mice, Nras G12D/+ lead to CMML in 95% of mice, in some of which the up-regulation of oncogenic Nras allele through uniparental disomy (UPD) was observed, and to acute T ALL in 8% of mice, in some of which up-regulation of Nras wild type allele was observed. Nras G12D/G12D expression restricted to BM compartment lead to 100% acute T ALL enriched with Notch1 aberrations [196].

The oncogenic potential of Nras Q61R aberration frequently observed in MM was also investigated. Nras Q61R/+ expression restricted to GC B cells lead to MM or other lymphoid diseases in only a minority of cases. On the contrary, Nras Q61R/+ expression in the context of indolent MM mouse model (Vκ*MYC) with concomitant activation of human MYC in GC B cells, resulted in high malignant MM with hyperactivation of the ERK and AKT pathways [198]. 

### 5.3. BRAF

The study of Braf V600E mutation in the murine hematopoietic compartment was prompted by the almost universal presence of this mutation in HCL cases, found in leukemic cells but also in the HSC and B progenitor cells of patients. The expression of Braf V600E in murine HSC resulted in a lethal disorder reminiscent of HCL, characterized by increased self-renewal capacity of early B lineage cells and impairment of myeloid and erythroid differentiation; the use of a BRAF inhibitor reversed the phenotype. Of note, the typical morphologic phenotype of hairy cells was not seen. On the contrary, the expression of Braf V600E in fetal hematopoietic cells resulted in embryonic lethality, whereas its expression in B lineage cells did not result in HCL or other malignant phenotypes, suggesting that specific alterations in the HSC compartment drive the HCL onset [200].

The Braf V600E aberration was also studied in the context of CLL. Braf V600E expression in B cells of Eμ-TCL1 mice, a well-established CLL mouse model, resulted in the acceleration of CLL onset and the shortening of mice survival. Braf V600E leukemia was characterized by reduced apoptosis and enhanced immune suppressive effects on the cells of the microenvironment. This model hints towards potential benefits of using RAF/MEK inhibitors and checkpoint inhibitors, and might be applied to test the activity of different drug combinatorial strategies [201].

## 6. Conclusions

KRAS mutations and mutations in the RAS-MAPK pathway show heterogeneous incidence among mature B cell neoplasms without apparent association with specific B cell differentiation stages or with specific signaling dependencies (e.g., BCR signaling or microenvironment). *KRAS*, *NRAS*, and *BRAF* are the most frequently mutated RAS-MAPK genes, with *KRAS* and *NRAS* aberrations being mostly mutually exclusive, and, when co-occurring, mainly affecting different subclones. *HRAS* is only rarely mutated, as observed in solid tumors and in line with the suggested function of a weak oncogene. As opposed to what was reported in T ALL and in other malignant contexts [202,203], no cooperation between NOTCH1 and RAS aberrations have been observed in mature B cell malignancies.

RAS-MAPK mutations are mostly considered late driver events in mature B cell neoplasms, found both clonally and subclonally, and are often associated with disease progression and drug resistance. The only notable exception is represented by *BRAF* V600E mutation in HCL, occurring early in the HSC compartment and driving the onset of disease. Accordingly, with the exception of Braf V600E, which proved to induce HCL-like disorder when expressed in the HSC compartment of transgenic mice, most of the RAS-MAPK genetic aberrations fail to induce B cell lineage oncogenic transformation when expressed in conditional murine models. On the contrary, when expressed in a malignant B cell context, RAS-MAPK genetic aberrations accelerate and sharpen the phenotype, as observed for Nras Q61R expression in the Vκ*MYC MM mouse model and Braf V600E expression in the Eμ-TCL1 CLL mouse model. This observation suggests that RAS-MAPK aberrations mostly need a cooperative genetic event to exert their oncogenic role in the onset and progression of B cell malignancies.

The clinical evaluation of RAS-RAF-MAPK inhibitors are advanced in some neoplasms, such as MM and HCL, while completely missing in others. The careful assessment of RAS-MAPK aberrations is not always a prerequisite in the use of RAS-RAF-MAPK inhibitors in the clinic, although it may help to anticipate response or resistance onset. Indeed, RAS-MAPK aberrations play a major role in MAPK pathway reactivation when inhibitors are used as single agents. Multi-agent therapies, like combining RAF/MEK inhibitors in HCL and MM, are appearing as a strategy to be preferred, achieving very promising results in clinical trials. 

In conclusion, while considered an exclusive hallmark of MM for many years, RAS-MAPK mutations turned out to be prominent players in the genomic landscape of mature B cell malignancies, affecting a consistent fraction of patients. As extensive genomic characterization improves, particularly in rare neoplasms, this fraction could further expand. Careful characterization is needed to fully elucidate the meaning of these aberrations in mature B cell neoplasms, also in view of the well-established role of KRAS aberrations in promoting an immunosuppressive and tumor protective microenvironment in the context of solid tumors.

Lastly, additional attention needs to be paid to genetic events involving *RAS*/*RAF* pseudogenes, frequently involved in copy number gains and transcriptional activation. The aberrant activation of the *Braf* pseudogene, proved to upregulate Braf and downstream MAPK pathways through competitive endogenous RNA mechanisms, was shown to induce B cell lymphoma (DLBCL) in mice [204].

Thus, in the bustling era for RAS-RAF-MAPK targeting molecules, the KRAS and RAS-MAPK signaling represents a crucial therapeutic target in mature B cell neoplasms that must be completely exploited.

## Figures and Tables

**Table 1:** RAS-MAPK pathway aberrations in mature B cell lymphoproliferative disorders.

	Gene (RefSeq Accession Number)										
Disease	KRAS	NRAS	HRAS	BRAF	RAF1	MAP2K1	MAP2K2	MAPK3	MAPK1	NF1	References
	(NM_033360)	(NM_002524)	(NM_001130442)	(NM_004333)	(NM_002880)	(NM_002755)	(NM_030662)	(NM_002746)	(NM_138957)	(NM_001042492)	
MCL	G12D, L19F	F156L		L485W, L485_V487del, N581I				V162L		G661fs	[9,10,11]
DLBCL	L6P, G12A/S/R/D, G13D, V14I, L19F, Q22K, L23R, E31K, T58I, Q61H, A83V, M111V, K117N, D119G/N, A146T, V160M, G174S (NM_004985), P178T, P178Vfs*2, C180X	G13D, I24N, Q61R, N116S, E153K	R41W, S145L, R153C/H (NM_176795), H166Y, P166S (NM_176795), R169Q, K170X	D22N, V120I, H223P, R260C, G327_splice, Q344L, R347Q, D352E, R360Q, N378S, R389H, R424Q, T440P, G466R, S467T, F468S, G469A, K483E, L485F, L525R, N581S/H, D594G/A/N, F595L, L597R/Q, T599R, V600E, K601E/N, R671Q, R719C	H105D, L149F, R333C, T353I, R391S, L476F, E478K	A26V, Q46L, R47Q, F53L/S/V/C/Y, K57T/E, D67N, A106T, C121S, N122D, Y130H/C, G176S, A283V, T292I, R291K, R305Q	Q60P, D71N, V202M	E367G, E203K	Y131H, I240V, E322A, A327S	S15N, Q28H, G57S, R156H, C245_E247del, R262H, Q282R, G312E, R681Q, R711H, E924X, R1066_splice, G1166_splice, I1186L, A1202S, R1276X, N1338Y, G1403S, R1462Q, V1674I, I1755V, A1858T, L1892X, H1962Pfs, I2026T, I2057V, I2127V, S2152T, T2204I, T2222A, I2267R, N2432S, V2511L, V2657_splice, Y2702F, P2717L, I2739T, Y2742S, T2773A, G2793E	[12,13,14,15]
FL						F53Y ^a^, Q56P ^a^, K57E/R ^a^, C121S ^a^			N297D ^a^, D321G ^a^	M1539Wfs*35	[16,17]
BL				E501A, T508I, D594G, L597R/Q	E478K		S248L				[18,19,20,21]
HL	L19F	V114L		D454G, S335Y D380_splice		L30F, L342X				C324S, R765C	[22,23,24]
CLL	V7G, G12D/C/V/S/R, G13D/C, V14I, A18V, L19F, Q22K/E, T50I, T58I, Q61H/L/R, K117N/R, A146T/V/P	G12C/D, G13D/R/V, S17T, Q61K/R/H/L, A146T		G466E, F468S, G469A/V/E/R, K483E, L485F, N486_P491del, E501K, G534R, N581S/I, D594G/N, F595L, L597R/Q, V600E, K601N/E, D638E	R391W	F53L/V/C, K57N, C121S, P124L, G128V/D, R201H, E203K	Q60P, Y134C		D291G	Y489C, T676fs, R1276Q, K1444N, R2594P	[25,26,27,28,29,30,31]
HCL	G12D, K117N	G12C		V600E, D449E, F468C, S602T		F53L, Q56P, K57E/T/N, I103N, C121S, L42_K57del					[32,33,34,35]
SMZL	E174K			V157I, V600E	R282X, S605F	K57E, F53L	R371Q			L1208F	[36,37,38,39]
NMZL				N58I, L597Q, V600E							[40]
EMZL-MALT			Y141C	Q709_splice						F2830L, I941V, W267X	[41,42]
SDRPL		G12V	V187A	G469A, K601Q	P261S	K57E/N, V60E, I103N, C121S					[38,43]
B-PLL				V600E							[44]
MM	G12A/V/D/C/R/S, G13D/V, V14I, A18D/V, L19F, Q22K/E, I24N, N26K, I36M, A59G, G60R, Q61H/L/R/K/P, E63K, Y64D/N, I84M, K117N/R, D119H, A146T/V, K147E	G12D/A/V/S/C/R, G13D/R/C/V, I46M, Q61H/L/R/K/P, Y64D/N, F82L, A91V, A146T, K147N		T241M, D287_splice, D380Y, G464V, G466E/V/A, S467L, G469V/A/R/E, K483Q, N486_T491 > K, I554T, N581S/I, E586K, D594G/N/H/E/A, G596V, L597Q, V600E, K601E		R47Q				T25K, Y49H, F51V, S168P, L1631fs*1, G1649E, S1938_splice, E2729fs*10	[45,46,47,48]
PBL	V7G, G12R/V/D, G13D/C, A59G, Q61H/R, E63K, K117N, D119N, A146T/V	G12D/R/V, G13D/C/R/V, A59D, Q61H/K/R, K117R, A146T	R123P, Q61K	G464E, G466V/R G469V/A/R, V471F, T599TT, V600E, K601N, M689V	S257L, V512E	K57N, C121S, P124R/L, T378N		M310Cfs*2	G16V	A2321D, S620I, A928S, R2637Q	[49,50,51,52]

For each gene, codons annotation refer to the RefSeq transcript listed on the top of the table, unless otherwise specified. ^a^ The reported mutations were identified in the pediatric-type follicular lymphoma (PTFL). MCL, mantle cell lymphoma; DLBCL, diffuse large B cell lymphoma; FL, follicular lymphoma; BL, Burkitt lymphoma; HL, Hodgkin lymphoma; CLL, chronic lymphocytic leukemia; HCL, hairy cell leukemia; SMZL, splenic marginal zone lymphoma; NMZL, nodal marginal zone lymphoma; EMZL-MALT, extranodal marginal zone lymphoma of mucosa-associated lymphoid tissue; SDRPL, splenic diffuse red pulp lymphoma; B-PLL, B cell prolymphocytic leukaemia; MM, multiple myeloma; PBL, plasmablastic lymphoma.

**Table 2:** RAS-MAPK pathway inhibitors in lymphoproliferative disorders.

Compound	Combination Therapy	Target Mechanism	Disease	Molecular Inclusion Criteria ^a^	Clinical Trial	Clinical Inclusion Criteria	Other Settings	References
vemurafenib	none	BRAF V600E inh	HCL	BRAF V600E	phase II: EudraCT 2011-005487-13 and NCT01711632	r/r	case report	[139,140]
			MM	BRAF V600E/K	phase II: NCT01524978	r/r	case report	[141,142,143,144,145,146]
	cobimetinib	BRAF V600E inh + MEK inh	HCL	BRAF V600E			case report	[147]
			MM	BRAF V600	phase II: NCT03297606	r/r	case report	[148,149]
			other B- NHL	BRAF V600	phase II: NCT03297606	r/r		NA
	rituximab	BRAF V600E inh + anti-CD20 mab	HCL	BRAF V600E	phase II: EudraCT-2014-003046-27	r/r		[150]
	obinutuzumab	BRAF V600E inh + anti-CD20 mab	HCL	BRAF V600E	phase II: NCT03410875	untreated		NA
dabrafenib	none	BRAF V600E/K inh	HCL	BRAF V600E	phase II: EudraCT-2014-001379-29	r/r		[151]
			CLL	RAS-MAPK			pre-clinical in vitro model	[29]
	trametinib	BRAF V600E/K inh + MEK inh	HCL	BRAF V600E	phase II: NCT02034110	r/r		[152]
			MM	KRAS, NRAS, BRAF	phase I: NCT03091257	r/r		NA
encorafenib	binimetinib	BRAF V600E/K/D inh + MEK inh	MM	BRAF V600E/K	phase II: NCT02834364	r/r		NA
TAK-580		pan-RAF inh	MM				pre-clinical in vitro model	[153]
sorafenib	none	RAF/multikinase inh	MM		phase II: NSC-724772	r/r		[154,155]
			CLL		phase II: NCT00303966; NCT01510756	r/r	pre-clinical in vitro model	[156,157,158,159]
			DLBCL		phase II: eastern cooperative oncology group study (E1404)	r/r	pre-clinical in vitro model	[160,161,162]
			HL				pre-clinical in vitro model	[163,164]
	everolimus	RAF/multikinase inh + mTOR inh	MM /other B-NHL		phase I/II: NCT00474929	r/r		NA
	bortezomid, R406	RAF/multikinase inh + Proteasome inh or Syk inh	MCL				pre-clinical in vitro and in vivo model	[165]
	rapamycin	RAF/multikinase inh + mTOR inh	FL/other B-NHL				pre-clinical in vitro model	[166]
	Givinostat	RAF/multikinase inh + HDAC inh	HL				pre-clinical in vitro and in vivo model	[167]
	perifosine	RAF/multikinase inh + AKT inh	HL/CLL		phase II	r/r	pre-clinical in vitro and in vivo model	[168,169]
AZD4785		antisense oligonucleotide targeting KRAS	MM	KRAS			pre-clinical in vitro model	[170]
CH5126766		MEK-pan-RAF inh	MM	KRAS and BRAF	phase I: NCT02407509	r/r		[171]
trametinib	none	MEK inh	HCL	MAP2K1			case report	[172]
			MM	KRAS, NRAS, BRAF		r/r	retrospective review of trametinib treated patients	[173]
	bortezomib	MEK inh + Proteasome inh	MM	NRAS			pre-clinical in vivo model	[174]
	tirabrutinib	MEK inh + BTK inh	DLBCL				pre-clinical in vitro model	[175]
cobimetinib	venetoclax, atezolizumab	MEK inh + BCL2 inh + anti-PD-L1 mab	MM		phase I/II: NCT03312530	r/r		[176]
binimetinib	ABT-737, venetoclax	MEK inh + BH3-mimetic or BCL2 inh	CLL				pre-clinical in vitro model	[177]
	MK2206, idelalisib	MEK inh + AKT inh or PI3K delta inh	CLL				pre-clinical in vitro model	[178]
selumetinib	none	MEK inh	MM		phase II: NCT01085214	r/r		[179]
			DLBCL		phase II: NCT01278615	r/r	pre-clinical in vitro and in vivo model	[180,181]
	LBH589, FK228	MEK inh + HDAC inh	MM	KRAS, NRAS, BRAF			pre-clinical in vitro model	[182]
pimasertib	ibrutinib, idelalisib	MEK inh + BTK inh or PI3K-delta inh	DLBCL/other B-NHL				pre-clinical in vitro model	[183]
UO126		MEK inh	HL				pre-clinical in vitro model	[184]
AEZS-136		PI3K/ERK dual inh	HL				pre-clinical in vitro and in vivo model	[185]
ulixertinib		ERK inh	CLL	RAS-MAPK			pre-clinical in vitro model	[29]
SCH772984	CI-1040, trametinib, idelalisib	ERK inh or MEK inh and/or PI3K- delta inh	CLL	MAP2K1			pre-clinical in vitro model	[102]

^a^ Molecular inclusion criteria refer to RAS-MAPK aberrations required for inclusion in the reported clinical trials, or mutations present in the in vivo/in vitro models used in the indicated pre-clinical studies. NHL, non-Hodgkin lymphoma; HL, Hodgkin lymphoma; HCL, hairy cell leukemia; MM, multiple myeloma; CLL, chronic lymphocytic leukemia; DLBCL, diffuse large B cell lymphoma; MCL, mantle cell lymphoma; FL, follicular lymphoma; HDAC, histone deacetylase; inh, inhibitor; NA, not available.

**Table 3:** Oncogenic RAS/RAF mouse models.

Target Gene	Mouse Model	Genetic Background	Model Type	Phenotype	Reference
KRAS	CMV-cre; LSL-KrasG12D	C57BL/6	Conditional KrasG12D expression in all tissues (mosaic pattern) at early embryonic stage	Embryonic lethality	[191]
	Mx1-Cre; LSL-KrasG12D	C57BL/6	Conditional KrasG12D expression in HSC	Development of MPD closely resembling CMML/JMML in all mice. Co-occurrence of T-ALL in minor fraction of mice. BM cell transplantation in primary recipient mice lead mostly to acute T-ALL enriched with Notch1 mutations	[192]
	Cγ1-Cre; LSL-KrasG12D	C57BL/6	Conditional KrasG12D expression in post-GC B cells	Development of thymic lymphomas and lung adenomas	[193]
	AID-Cre-YFP; LSL-KrasG12D	129/SvJ x C57BL/6	Conditional KrasG12D expression in B cells undergoing GC reaction	No hematopoietic phenotype, development of focal epidermal papillomas	[193]
	AID-Cre-YFP; LSL-KrasG12D; Arf-/-	129/SvJ x C57BL/6	Conditional KrasG12D expression in B cells undergoing GC reaction in the context of tumor-prone *Arf*-null background (KRAS cooperating mutation).	Impairment of splenic architecture with deficiency of GC, increased polyclonal antibody responses over time. Development of fatal epidermal papillomas and cutaneous sarcomas	[193]
	Mx1-Cre; LSL-KrasA146T	C57BL/6	Conditional KrasA146T expression in HSC	Development of MDS/MPN with expansion of immature myeloid cells in the BM and spleen. Delayed disease onset and death in comparison to Mx1-Cre; KrasG12D mice	[194]
	Kras+/V14I or KrasV14I/V14I	129S2/Sv, C57BL/6J or mixed B6/129	Constitutive KrasV14I expression	Noonan syndrome phenotype and development of MPD reminiscent of human JMML in KrasV14I/V14I; milder phenotype in the heterozygous model	[195]
NRAS	Mox2-Cre/+; LSL-NrasG12D/+	C57BL/6	Conditional NrasG12D expression in epiblasts beginning at E5	Embryonic lethality	[196]
	Mox2-Cre/+; LSL-Nras G12Dhypo/+ or LSL-Nras G12Dhypo/G12Dhypo	C57BL/6	Conditional NrasG12D hypomorphic allele expression (equivalent to 25–40% of single copy Nras wild type allele) in epiblasts beginning at E5	No hematopoietic phenotype	[196]
	Mx1-Cre; LSL-NrasG12D/+ or LSL-NrasG12D/G12D	C57BL/6	Conditional NrasG12D expression in HSC	In the homozygous model, development of acute MPD with ERK hyperactivation at 12 months. BM cells transplantation in primary recipient mice lead to 100% acute T-ALL enriched with Notch1 mutations; milder phenotype in the heterozygous model: development of histiocytic sarcoma (predominant) or chronic MPD (occasional) resembling CMML at 12 months. BM cells transplantation in primary recipient mice lead to 95% CMML and 8% acute T ALL.	[196,197]
	IgG1-Cre; LSL-NrasQ61R/+	C57BL/6	Conditional NrasQ61R expression in GC B cells	Development of MM or other lymphoid disease in a fraction of mice	[198]
	Vκ*MYC; IgG1-Cre; LSL-NrasQ61R/+	C57BL/6	Conditional NrasQ61R expression in GC B cells of Vκ*MYC mice (indolent MM mouse model)	Development of highly malignant MM characterized by high proliferation index, hyperactivation of ERK and AKT signaling, impaired hematopoiesis, extramedullary disease and expression of human MM gene signatures.	[198]
BRAF	CMV-Cre; LSL-BrafV600E	C57BL/6	Conditional BrafV600E expression in all tissue (mosaic pattern) at early embryonic stage	Embryonic lethality	[199]
	Vav-cre; LSL-BrafV600E	C57BL/6	Conditional BrafV600E expression in prenatal hematopoietic cells	In utero hematopoietic transformation and embryonic lethality beyond day 12.5	[200]
	Mx1-cre; LSL-BrafV600E	C57BL/6	Conditional BrafV600E expression in HSC	Development of HCL-like disorder characterized by extramedullary hematopoiesis, impaired erythroid differentiation, increased clonogenic capacity of B lineage cells and increased circulating soluble CD25. No hairy cells morphologic phenotype	[200]
	Cd19-cre; LSL-BrafV600E	C57BL/6	Conditional BrafV600E expression in B lineage cells	No hematopoietic phenotype. MAPK signaling activation in B lineage cells, minimal elevation of soluble CD25	[200]
	Eµ-TCL1; Cd19-cre; LSL-BrafV600E		Conditional BrafV600E expression in B lineage cells of Eµ-TCL1 mice (CLL mouse model)	Acceleration of CLL onset with decreased spontaneous apoptosis, enhanced immune suppression and shortening of mice survival	[201]

HSC, hematopoietic stem cells; BM, bone marrow; GC, germinal center; MPD, myeloproliferative disorder; CMML, chronic myelomonocytic leukemia; JMML, juvenile myelomonocytic leukemia; ALL, acute lymphoblastic leukemia; MDS, myelodysplastic syndrome; MPN, myeloproliferative neoplasm; MM, multiple myeloma; HCL, hairy cell leukemia; CLL, chronic lymphocytic leukemia.
